# Heterogeneous Mental Health Responses to the COVID-19 Pandemic in Germany: An Examination of Long-Term Trajectories, Risk Factors, and Vulnerable Groups

**DOI:** 10.3390/healthcare11091305

**Published:** 2023-05-03

**Authors:** Malvika Godara, Jessie Rademacher, Martin Hecht, Sarita Silveira, Manuel C. Voelkle, Tania Singer

**Affiliations:** 1 Social Neuroscience Lab, Max Planck Society, 10557 Berlin, Germany; jessie.rademacher@gv.mpg.de (J.R.); sarita.silveira@social.mpg.de (S.S.); singer@social.mpg.de (T.S.); 2 Department of Psychology, Helmut Schmidt University, 22043 Hamburg, Germany; martin.hecht@hsu-hh.de; 3 Institute of Psychology, Humboldt University of Berlin, 10117 Berlin, Germany; manuel.voelkle@hu-berlin.de

**Keywords:** mental health, vulnerability, resilience, COVID-19, pandemic, trajectories

## Abstract

Abundant studies have examined mental health in the early periods of the COVID-19 pandemic. However, empirical work examining the mental health impact of the pandemic’s subsequent phases remains limited. In the present study, we investigated how mental vulnerability and resilience evolved over the various phases of the pandemic in 2020 and 2021 in Germany. Data were collected (n = 3522) across seven measurement occasions using validated and self-generated measures of vulnerability and resilience. We found evidence for an immediate increase in vulnerability during the first lockdown in Germany, a trend towards recovery when lockdown measures were eased, and an increase in vulnerability with each passing month of the second lockdown. Four different latent trajectories of resilience–vulnerability emerged, with the majority of participants displaying a rather resilient trajectory, but nearly 30% of the sample fell into the more vulnerable groups. Females, younger individuals, those with a history of psychiatric disorders, lower income groups, and those with high trait vulnerability and low trait social belonging were more likely to exhibit trajectories associated with poorer mental well-being. Our findings indicate that resilience–vulnerability responses in Germany during the COVID-19 pandemic may have been more complex than previously thought, identifying risk groups that could benefit from greater support.

## 1. Introduction

The spread of the Coronavirus Disease 2019 (COVID-19) since early 2020 has created significant health and socioeconomic challenges for global society. Crucially, the pandemic and the related lockdowns imposed across the world to curb the spread of the disease have come to be seen as a severe and sustained stressor that has left us grappling with psychological consequences [1]. Initial cross-sectional studies documented high levels of psychological distress, depression, anxiety, and worry as an immediate response to the declaration of pandemic and confinement measures while also indicating low levels of resilience (see reviews [2,3,4,5]). Significant increases in mental health challenges were corroborated by existing cohort studies and longitudinal studies in the early months of the pandemic [6,7,8,9,10,11,12,13,14,15,16,17]. However, these initial studies could not account for longer-term changes in the pandemic trajectory and the mental health response to changing pandemic features, such as second lockdowns or the introduction of vaccination programs. Therefore, in the present study, we sought to investigate how mental health evolved over the longer course of the pandemic in Germany in 2020 and 2021, during the various phases of lockdowns and deconfinement periods. To provide a more comprehensive picture, we focused on changes in multiple aspects of mental health, such as depression, anxiety, loneliness, coping behavior, pandemic-related mental burdens, psychosomatic complaints, stress recovery, and life satisfaction, to name a few.

After the initial COVID-19 pandemic-related lockdowns in March 2020, several successive waves of the pandemic led in many countries to repeated lockdowns, requiring classroom closure, remote work, confinement, and physical distancing, such as the second longer and more gradual lockdowns in Germany starting in November 2020 and lasting until April 2021. This re-tightening of safety measures after a summer of deconfinement could have led to further disturbances in the mental health of different segments of the population. Initial evidence from empirical studies indicates significant deterioration of mental health in the second lockdowns [18,19]. Moreover, a small number of studies that examined the longitudinal trajectories of mental health during 2020 and 2021 found poor mental health outcomes in several different sub-groups of people [20,21,22,23,24,25]. In particular, few of these longitudinal studies showed that levels of mental health problems tended to increase with each measurement occasion [26,27], which is in line with cumulative risk models [28,29,30]. This points to a unique feature of the COVID-19 pandemic, such that the longer individuals were under strict lockdown conditions, the worse their mental health became, leading to what can be termed a pandemic fatigue effect [31].

The concept of the pandemic fatigue effect, initially introduced to account for increasing demotivation to adhere to mandated safety measures observed in the general population [32], has also been theorized in the context of mental health by a recent conceptual model of psychological resilience and vulnerability during the COVID-19 pandemic, the Wither or Thrive Model of Resilience (With:Resilience) [31]. The With:Resilience model proposed that the different phases of the pandemic are likely to have led to distinct resilience–vulnerability responses. Conceptualizing mental health difficulties on a bipolar spectrum, ranging from vulnerability on one end to resilience on the other, the model posited two specific effects. First, the model postulated an acute stressor effect resulting from the introduction of the first confinement measures leading to an immediate increase in mental health problems seen in the form of greater psychological vulnerability. After the lifting of the lockdown and confinement measures, the model proposes that the general population is likely to demonstrate a tendency towards the resilience end of the spectrum, i.e., reduced mental health difficulties. The model then proposes a second effect owing to the long-term stress of repeated confinement and lockdowns, contextualizing pandemic fatigue from a mental health perspective. Several studies have documented the initial acute stressor effect and the recovery of mental health difficulties in the early phases of the pandemic [13,14,15,16,17,33]. However, the pandemic fatigue effect on mental health has remained understudied [34,35], with only some early evidence of this phenomenon being observed in some recent studies from Argentina and the UK [26,27]. As such, the full range of resilience–vulnerability responses concerning the different phases of the pandemic, such as repeated lockdowns, remain poorly understood so far in many countries, including Germany. Therefore, the first aim of the present study was to examine pandemic-related mental health changes in Germany in 2020 and 2021 and investigate acute stressor and pandemic fatigue effects.

Additionally, it is expected that individual differences would likely lead to further unique patterns of pandemic-related mental health trajectories being observed during the pandemic. The With:Resilience model proposed that four different trajectories of resilience–vulnerability (chronic vulnerability, cumulative vulnerability, resilient recovery, and non-reactive resilience) would be observed as a function of the various phases of the pandemic. This view is also in line with prevalent models of post-traumatic mental health trajectories [36,37]. In support of this view, several studies in the early phases of the pandemic identified several trajectories of mental health, the number differing across studies [7,13,26]. A recent study from Argentina, covering a longer period of the pandemic, found trajectories of mental health that were distinct from those observed in previous studies examining only shorter time periods [26]. As such, it remains to be seen what kind of different resilience–vulnerability time courses would emerge in the German population when considering a longer course of the pandemic timeline. The second goal, therefore, was to identify unique resilience–vulnerability trajectories covering multiple measurement occasions over a longer duration (>12 months) of the pandemic.

Another important goal of the present study was to understand if there are specific sociodemographic and trait psychological factors that predict heterogeneous responses, i.e., which display unique mental health trajectories. Unique individual and contextual factors are likely to enhance or inhibit the probability of an individual exhibiting a certain trajectory. The With:Resilience model posits several key categories of factors, such as individual psychological and biological or social intersubjective factors, that could influence which mental health trajectory an individual exhibits over the course of the pandemic. In line with this proposition, several studies investigating resilience–vulnerability trajectories during the early phases of the pandemic have delineated a variety of predictors [7,13,15,26,38,39]. For example, demographic factors such as sex, age, employment status, education and income levels, and trait characteristics such as neuroticism and pessimism have consistently emerged as key predictors of mental health both before and during the pandemic [40,41,42,43]. Accordingly, the third aim of the present work was to identify demographic factors and other enduring psychological trait aspects that serve as risk factors for the different longitudinal trajectories of resilience–vulnerability in our study.

**Figure 1:**
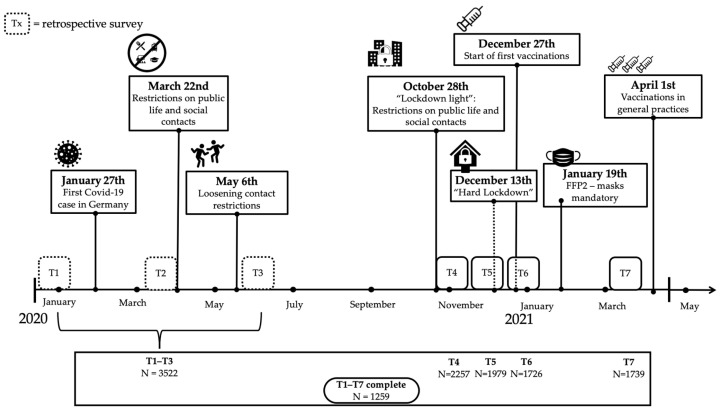
Timeline of phase 1 of the CovSocial project. The seven measurement occasions (T1–T7) are presented in the context of various COVID-19 pandemic-related developments in Germany in 2020 and 2021. Sample size at each assessment is also provided.

To investigate these questions, we relied on the data from the CovSocial project, which is a longitudinal investigation of the effects of the COVID-19 pandemic in 2020 and 2021 on various biopsychosocial aspects in a large cohort of Berliners [44]. In the first phase of the CovSocial project, participants reported on several aspects of vulnerability and resilience, such as depression, anxiety, optimism, and the use of coping strategies. Repeated measurements were taken over seven unique measurement occasions during the pandemic course in Germany: covering the pre-pandemic timepoint in January 2020, the first lockdown in March–April 2020, deconfinement and reopening period in May–September 2020, the “lockdown light” in October 2020, and three timepoints over the second “hard lockdown” in November 2020–March 2021 (see Figure 1).

In a previous study reporting on the data from the first three timepoints [11], a unique bipolar resilience–vulnerability latent factor emerged that incorporated multiple state indicators of vulnerability and resilience, with positive factor loadings for vulnerability indicators and negative factor loadings for resilience indicators. This is in line with the theoretical conceptualization of resilience and vulnerability as complementary counterparts [45,46]. Using this composite bipolar latent resilience–vulnerability factor, Silveira et al. [11] showed a trend towards increased vulnerability during the lockdown period and a trend towards resilience upon re-opening and easing of lockdown measures. A drawback of many of the studies examining mental health trajectories during the pandemic so far is the use of single measures that often only account for limited aspects of psychological vulnerability or resilience, such as the use of the General Health Questionnaire [7,13]. The With:Resilience model postulates that by over-reliance on one single measure, the risk of failing to capture unique aspects of vulnerability becomes pronounced. Aspects which gained particular salience during the pandemic, such as loneliness resulting from social isolation or mental burdens emerging from multi-tasking due to school closures, have been largely ignored in the determination of resilience–vulnerability profiles in the studies published so far. The approach adopted by Silveira et al. [11] counters this limitation, providing a more comprehensive picture. Therefore, in the present study, we took the same approach as that implemented by Silveira et al. [11] and constructed a composite bipolar resilience–vulnerability latent factor at each of the seven timepoints. We then used these factors to investigate the three aims of the present work.

First, we scrutinized the temporal dynamics of the resilience–vulnerability latent factor over the seven timepoints to chart out the general course of mental health. Importantly, we investigated whether an “acute stressor effect” and a “pandemic fatigue effect” can be observed in the general mental health time course during the two national lockdowns in Germany. Second, we explored the presence of unique heterogeneous trajectories of resilience–vulnerability and whether these are in line with the theoretical predictions of the With:Resilience model. Finally, we examined whether certain demographic factors, such as sex, age, and income, and trait psychological aspects, such as neuroticism, pessimism, and empathy, can predict which trajectory of resilience–vulnerability is exhibited by an individual. This will allow a more differentiated and nuanced understanding of the impact of the pandemic on mental health, leading to the identification of risk factors and specific vulnerable groups.

## 2. Materials and Methods

### 2.1. Sample

The current study is part of the multi-phase CovSocial project. The first phase of the project examined the impact of the pandemic-related lockdowns in Germany on various biopsychosocial domains, including vulnerability, resilience, and social cohesion, in a sample from Berlin, Germany. The second phase of the project focused on the efficacy of app-delivered interventions for mental health and social capacities. The present study uses data from the first phase of the project. The sample for the current study, recruited from the general Berlin population, includes 3522 participants aged 18–65 years (mean age = 43.95 ± 12.69 years, 65.11% female). Table 1 provides an overview of the sample characteristics.

Participants for the CovSocial study were recruited during the period August 2020 to November 2020 using a variety of recruitment methods, such as sending 56,000 letters to addresses that were randomly selected by the residents’ registration office in Berlin, e-mail lists of academic and research institutions, flyers at churches and sports clubs, social media postings, as well as advertisements in newspapers and on public transportation. Initially, 7214 individuals signed up to participate in the study. Eventually, only 3681 individuals completed the first survey comprising demographic and trait questionnaires, as well as the state-level questionnaires for the first three retrospective timepoints. Participants were excluded from the study if they did not meet the inclusion criteria: age between 18 and 65 years (n = 81) and residing in Berlin (n = 44). Participants were also excluded based on their speed of filling out the questionnaires, i.e., too fast or merely clicking through the questionnaire (n = 30), and content-based inconsistencies in demographic questions (n = 4). This led to a final sample of 3522 participants. Supplement File S1 provides further information on the recruitment and exclusion of participants.

Initially, the first phase of the project was planned as a retrospective assessment of psychosocial factors during the first pandemic-related lockdown in Germany and the periods preceding and following it (T1–T3; Figure 1). However, due to the dynamic nature of the pandemic, the study was extended to cover not only the first German lockdown but also the second wave of slowly increasing lockdown restrictions and the introduction of the vaccination program in 2020 and 2021. Therefore, participants who completed all questionnaires at the first three retrospective timepoints were invited to answer monthly follow-up questionnaires at four further timepoints (T4–T7). Given the extended nature of the assessment, we witnessed longitudinal drop-out during this second period of data acquisition (see Figure 1). In the analysis, this was dealt with through multiple imputations for missing data at T4–T7 timepoints (see Analysis section). All participants provided written informed consent before participation. The study was approved by the Ethics Committee of Charité—Universitätsmedizin Berlin (#EA4/172/20 and #EA1/345/20) and was conducted in accordance with the Declaration of Helsinki.

### 2.2. Measures

Resilience–vulnerability measures consisted of both validated scales and self-generated questions. Stress perception was measured by the short version of the Perceived Stress Scale (PSS-4; [47]), using a sum score of the four items. Depressive symptoms were assessed using the Patient Health Questionnaire-2 (PHQ-2; [48]), and anxiety symptoms using the Generalized Anxiety Disorder Scale (GAD-2, [49]), each by summing the respective two items. Moreover, beliefs about self-efficacy were assessed by the General Self-Efficacy Short Scale (ASKU; [50]), using the mean score of the three items. Self-generated questions were developed specifically for capturing aspects of resilience–vulnerability that are specific to the given pandemic and its dynamic nature, including pandemic-related burdens, psychosomatic complaints, loneliness, stress recovery, coping approaches, optimism, life satisfaction, and the perception of the pandemic as a chance or an opportunity for self and society (positive reappraisal of the pandemic). All self-generated questions were answered using a 9-point Likert scale. A full list of these questions and their mean values at each of the 7 timepoints can be found in the Supplementary Information (Files S2 and S3). All questionnaires and self-generated questions were presented in German, either by using the validated German form or by translating them for the study.

### 2.3. Study Design

Data for the present study were collected with repeated online surveys of the state measures mentioned above, administered through the CovSocial web app (www.covsocial.de (accessed 24 February 2023)). Assessments took place at seven timepoints: T1 (before lockdown in January 2020), T2 (during the first lockdown from mid-March to mid-April 2020), T3 (in June 2020 when restrictions were eased), T4 (November 2020), T5 (December 2020), T6 (January 2021), and T7 (mid-March to mid-April 2021). The first three timepoints were assessed retrospectively, in three separate blocks of questionnaires, from 11 September 2020 to 7 December 2020. During this retrospective phase, participants were asked to respond to questions taking the perspective of the particular timeframe. For example, when responding to questions in the T1 block, participants had to respond to how they felt and behaved during January 2020. For the last four timepoints (T4–T7), participants answered the blocks of questionnaires at the end of each month and rated their feelings, perceptions, and behavior for the last four weeks. As part of the online survey for the retrospective period, participants also completed blocks of demographic and trait measures. Trait measures comprised validated psychological questionnaires assessing various time-stable aspects of trait resilience-vulnerability, adaptive capacities, social belonging, and social capacities (Chronic stress, neuroticism, trait anxiety, pessimism, trait loneliness, trait maladaptive emotion regulation styles (self-blame and catastrophizing), trait stress recovery, trait self-compassion, trait life satisfaction, trait optimism, trait adaptive regulation styles (such as active coping and positive reframing), trait trust, trait social support, trait prosocial behavior, trait empathy, and trait perspective taking. For further details on trait questionnaires that were used to previously formulate the latent trait factors, please see Silveira et al. [51]). These trait measures, assessed as part of the CovSocial project, have been previously validated as latent trait factors and previously published [51]. In the present study, the estimated factor scores of these trait latent factors are being used for the first time to assess trait influence on state resilience–vulnerability trajectories.

### 2.4. Data Analysis

Statistical analysis was performed in four steps: (1) missing data imputation, (2) measurement model and invariance analyses, (3) growth mixture modeling, and (4) multinomial regression. R (v4.0.3; [52]) with packages mice (v3.13.0; [53]) and lavaan (v0.6-11; [54]) was used for the first two steps, and Mplus (v8; [55]) for the last two steps. All analyses that included significance testing were performed with a significance level of α = 0.05.

#### 2.4.1. Missing Data Imputation

While only complete data were used from T1 to T3, data of entire measurement timepoints were missing for some persons due to the extended longitudinal design in timepoints T4 to T7. Additionally, to avoid unreliable data, we removed an individual’s measurement occasion if a participant answered at least 2 out of 8 control questions (‘please set the slider on 3’) incorrectly, which was the case for n = 3 participants in T4, T6 and T7, respectively. We imputed missing data using predictive mean matching for all numeric variables through the multivariate imputation by chained equations (mice) method [56]. Trait, demographic, and repeated state measures served as predictor variables for the imputation model. A variable was only used as a predictor if the proportion of usable cases was at least 0.25 and the correlation with the to-be-imputed variable was higher than 0.25. An average of 20 predictor variables for each imputed variable was used. In total, 21 fully imputed data sets were used for further analyses.

#### 2.4.2. Measurement Model

**Figure 2:**
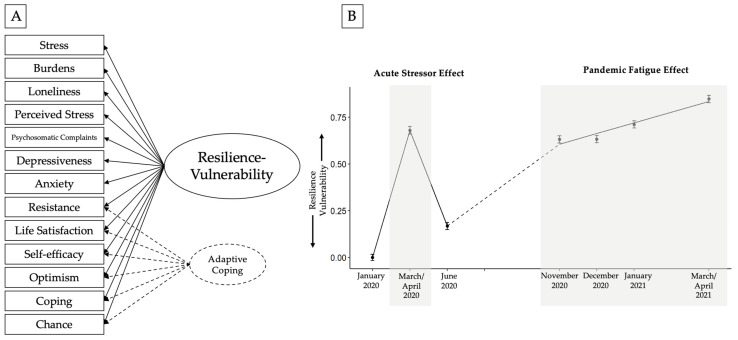
Panel (**A**) presents the latent factor model with the various indicators of resilience–vulnerability that entered the model. It also shows the additional adaptive coping factor that was modeled at each of the seven timepoints, which captured residual variances of the resilience indicators. However, this additional factor is not used for further analyses in this paper due to the low factor score reliabilities for this factor at all seven timepoints and also due to the focus of the current paper on more classic resilience–vulnerability aspects. Panel (**B**) presents the resilience–vulnerability growth trajectory across the seven timepoints, with acute lockdown and pandemic fatigue effects being observed during two lockdowns. Grey panels indicate the two lockdown periods in Germany.

For each of the seven timepoints, we specified a latent resilience–vulnerability factor using confirmatory factor analyses (see Figure 2A). The measurement model was adapted from Silveira et al. [11], where data from the first three retrospective timepoints were used to examine different latent factor models, including a latent resilience–vulnerability factor. In the present study, we also modeled such a bi-polar factor comprising vulnerability indicators of stress, burdens, loneliness, perceived stress, psychosomatic complaints, depressive symptoms, and anxiety, as well as resilience indicators of resistance, life satisfaction, self-efficacy, optimism, coping, and crises as a chance. Moreover, an additional adaptive coping factor was modeled at each of the seven timepoints, in line with Silveira et al. [11], which captured residual variances of the resilience indicators. However, while the residual adaptive coping factor was specified in the measurement model, it was not used for further analyses because this paper focuses on classic aspects of the resilience–vulnerability construct.

To ensure that means of the latent factors are comparable across measurement occasions, constraints of scalar measurement invariance (i.e., equality of factor structure, factor loadings, as well as intercepts across measurement timepoints) were introduced. Due to the differences in data acquisition between T1–T3 and T4–T7, and because no direct comparison of mean values between the two periods was intended, factors of T1 to T3 and factors of T4 to T7 were constrained to measurement invariance separately, that is, parameters were set equal only within each period. However, all timepoints, regardless of different constraints, were modeled in the same structural equation model with the factor mean of T1 set to 0 and factor variance of T1 set to 1. Thereby, we tried to enable the descriptive presentation of the whole trajectory and a relative level of all timepoints compared to the baseline.

Maximum likelihood estimation was used for parameter estimation. The analysis was repeated for each imputed data set, and results were pooled across analyses afterward. Model fit indices indicated an acceptable fit of the scalar measurement invariance model with CFI = 0.93, TLI = 0.93, and RMSEA = 0.026. In the last step, we estimated factor scores of the main resilience–vulnerability factor for each participant and each imputed data set using the standard method. The reliability of the estimated factor scores was reasonably high (ranging between 0.91 and 0.95 for each of the seven timepoints averaged over multiple imputations). As mentioned previously, the residual adaptive coping factor was specified in the measurement model but not used for further analyses due to the focus of the paper on classic aspects of the resilience–vulnerability construct. Moreover, the factor score reliability of the residual factor was considered too low for using these factor scores for further analyses (ranging between 0.20 and 0.30 for each of the seven timepoints averaged over multiple imputations).

#### 2.4.3. Growth Mixture Modeling

Growth mixture modeling was accomplished in two steps. First, to examine the general trajectory of resilience–vulnerability over time, the seven extracted factors were modeled using latent change score (LCS) and growth curve modeling techniques in Mplus for each imputation, respectively. Changes in resilience–vulnerability from T1 to T2 (acute lockdown effect) and from T2 to T3 (effect of re-opening) were assessed by modeling the differences between the respective timepoints as latent variables, referred to as LCS1 (T1 to T2) and LCS2 (T2 to T3) in the following. The average amount of change is reflected in the mean of each change score; the variance informs about the extent of inter-individual differences in the amount of change. This latent change score analysis approach has been previously applied by Silveira et al. [11] to T1–T3 data. Furthermore, we freely estimated the covariance between LCS1 and resilience–vulnerability at T1, as well as between LCS2 and resilience–vulnerability at T1, to account for the assumed correlation between change scores and baseline state factor. To capture the expected increase in mental burdens due to the increasingly intensified lockdown restrictions in Germany, a linear growth function was specified for T4 to T7. The latent slope factor was defined by factor loadings of 0 at T4, 1 at T5, 2 at T6, and 4 at T7 since the time elapsed between measurements was one month between T4, T5, and T6 and two months between T6 and T7. Thus, the latent intercept factor was positioned at T4.

In the second step, a mixture analysis was applied to the growth model to examine whether there are different latent classes of resilience–vulnerability time courses. The method of growth mixture modeling is used to identify unobserved subpopulations with different mean growth trajectories [57]. This is realized as a latent categorical variable on which the growth parameters (in our case, latent change scores and intercept and slope of the growth curve) are regressed. All parameters, including means and (residual) variances of growth parameters and latent factors, were allowed to vary freely between classes. To identify the optimal number of classes, we iteratively increased the number of classes in the model, and compared model fit in terms of Akaike’s Information Criterion (AIC) and Bayesian Information Criterion (BIC). A lower AIC and BIC represent a better fit. Furthermore, we considered the entropy (i.e., classification accuracy) of the different models and evaluated whether an additional class led to a superior solution in terms of theoretical plausibility (e.g., mean trajectories, number of assignments per class).

#### 2.4.4. Multinomial Logistic Regression

To identify trait and demographic factors that predict class membership, we followed the Three-Step Approach of Vermunt [58] to include (auxiliary) predictor variables in a mixture model [59]. According to that, after the specification of the mixture model without including predictors (step 1, already described in the previous section), the participants were assigned to the latent classes based on their posterior class membership probabilities (step 2), and these class assignments were used as dependent variables in a multinomial regression (step 3). We implemented this by using the R3STEP method in Mplus.

We introduced the following predictors into the multinomial regression model: trait resilience-vulnerability, trait adaptive capacities, trait social belonging, trait social capacities, age, (female) sex, psychiatric diagnosis, civil status (being married, cohabiting or in a partnership), household income (lower than Berlin monthly average net income €2175 [60]), full-time employment, years of education, and migration background. The first four trait predictors mentioned above refer to latent trait factors, which were developed in an earlier study of the CovSocial project [51]. These latent trait factors comprise trait indicators, such as neuroticism and pessimism. For further details on questionnaires and measures that the trait factors comprise, see Silveira et al. [51]. For each of the four trait factors, we estimated factor scores for every participant applying the standard method, and these factor scores were used in our analysis. The reliability of the estimated factor scores ranged between 0.93 and 0.51 for the four trait factor scores.

## 3. Results

The growth trajectory model had a very good pooled model fit (CFI = 0.99, TLI = 0.98, RMSEA = 0.065), indicating that this model is a good representation of the resilience–vulnerability time course of the entire sample. A depiction of the modeled trajectory can be seen in Figure 2B. Results show that there is a significant mean increase in resilience–vulnerability from T1 to T2 (acute stressor effect) of 0.68 (*p* < 0.001) and a significant mean decrease in resilience–vulnerability from T2 to T3 (reopening effect) of −0.51 (*p* < 0.001) in the general population. Further, an estimated growth curve slope of 0.057 (*p* < 0.001) indicates that there is a steady linear increase in resilience–vulnerability during the second lockdown. Descriptively, one can see that the mean value at T7 (0.847) even exceeds the peak of the acute lockdown effect in T2 (0.680). The intercept of the growth curve part, representing the mean value at T4, is significant with a value of 0.60 (*p* < 0.001). Likewise, the variances of the latent change factors and the latent linear growth factor are significant (Var(LCS1) = 0.937, *p* < 0.001; Var(LCS2) = 0.493, *p* < 0.001; Var(slope) = 0.009, *p* < 0.001). As such, we observe an acute stressor effect from T1 to T2 and a recovery effect from T2 to T3. Meanwhile, from T4 to T7, a pandemic fatigue effect is observed, and descriptively the levels of vulnerability from 2020 to 2021 seem to have worsened.

In the next step, we examined whether the differences between individuals could be due to different latent classes. Mixture analyses with different numbers of classes revealed that model fit improved up to a 5-class solution (see Table 2). A 6-class solution could not be estimated due to convergence issues. The additional gain in fit from the 4-class to the 5-class solution was, however, rather small (0.48% in AIC, 0.14% in BIC). Moreover, the smallest class in the 5-class solution had only 5.5% of the sample, which is on the verge of the recommended sample size [61], and class distribution of the 5-class solution was not theoretically more plausible and more informative because the growth patterns of classes 2 and 3 were very similar and close to each other. In contrast, the 4-class solution represented the most plausible solution based on class distribution, plotting of trajectory patterns, and theoretical assumptions and was, thus, retained as the final solution. The entropy of this model was 0.64, which is below the often-suggested threshold of 0.80 [62]. However, the average latent class posterior probabilities, representing another index of model classification accuracy, were above or close to the common threshold of 0.80 (see Table 3; [62]). Therefore, the obtained classes were assumed as well-separable, serving as a good basis for further analyses.

**Figure 3:**
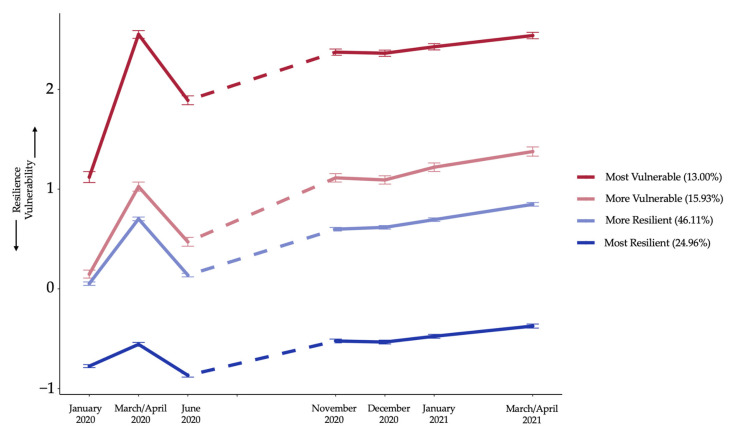
The observed resilience–vulnerability time courses of the four classes. Using a growth mixture analysis, final class counts and proportions for the latent classes based on their most likely latent class membership were as follows: “Most Vulnerable” class 1 = 450, “More Vulnerable” class 2 = 587, “More Resilient” class 3 = 1622, “Most Resilient” class 4 = 863.

In the 4-class model, descriptively, all classes showed a similar growth pattern comparable to the time course of the overall population but at different levels (see Figure 3). Importantly, in change scores, it can be seen descriptively that in classes 1 and 2, recovery after the first lockdown was not to levels prior to the lockdown, while in classes 3 and 4, recovery to baseline levels could be observed. Along these lines, a descriptive comparison of the slope from T4 to T7 shows the steepest increase in vulnerability in class 2 and the least in class 4. Therefore, in the following, we refer to class 1 as the most vulnerable class, class 2 as more vulnerable, class 3 as more resilient, and class 4 as the most resilient. All means and variances of the estimated parameters were significantly different from zero, indicating a significant mean increase from T1 to T2, a significant mean decrease from T2 to T3, as well as a significant mean linear increase from T4 to T7 in each class, and significant between-person variation in these effects. All parameter estimates can be seen in Table 4.

To examine how the growth patterns differed between classes and which growth parameters were most important to distinguish classes beyond the resilience–vulnerability level at baseline (i.e., T1), we tested change scores, intercept, and slope of each class for significance using a Wald test. First, a joint test of equality of the respective parameter for all classes was conducted. Second, we tested post-hoc paired comparisons to give a more detailed picture of which classes’ parameters differ significantly, with a Bonferroni corrected α of 0.0083 (due to 6 comparisons for each parameter). A Wald test of parameter constraints revealed that, overall, both latent change scores, as well as the slope differed significantly between classes. Post-hoc tests, however, showed that the LCS1 mean of the most resilient class significantly differed from the estimates of all other classes, and the LCS1 mean of the more resilient class differed significantly from the estimate of the most vulnerable class. Moreover, the LCS2 mean of the most resilient class significantly differed from the LCS2 mean of all other classes. For growth curve slopes, only the comparison of slopes of the more vulnerable and the most resilient class was significant. The result statistics of these post-hoc tests can be seen in Table 5.

**Figure 4:**
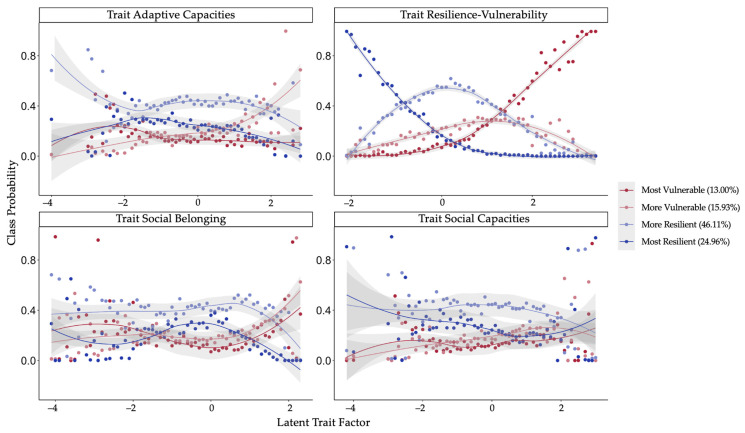
Probability of individual membership in a certain class as a function of the level of the latent trait predictors. The latent trait predictors used in the multinomial regression model were trait resilience-vulnerability, adaptive trait capacities, trait social belonging, and trait social capacities. The dots represent the predicted probabilities, and the lines depict the smoothed curves.

In the final step, we assessed the importance of various trait and demographic predictors in explaining class membership using a multinomial regression procedure. Odds ratios for the included predictors were calculated in comparison to a reference class. We set the most resilient class as the reference group, which means that the reported odds ratios reflect the chance of being in a specific other latent class as opposed to the most resilient group (see Table 6). Amongst trait predictors, trait resilience–vulnerability emerged as the most robust predictor of all classes, such that there were greater odds of individuals with higher levels of trait vulnerability being in the most vulnerable class, followed by the more vulnerable and then the more resilient class (see Figure 4). In a similar vein, there were greater odds of individuals with lower levels of trait social belonging being in a more vulnerable class. On the other hand, we observed greater odds of being in the more vulnerable class with higher levels of trait adaptive capacities followed by the more resilient class. Trait social capacities did not emerge as a significant predictor for class assignments.

**Figure 5:**
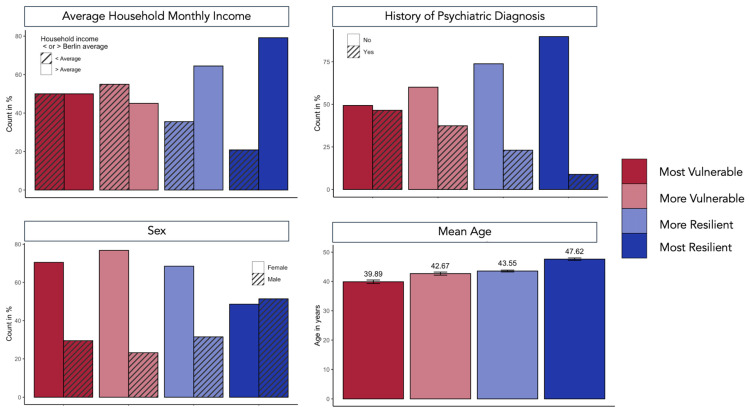
The demographic characterization of the four classes according to four demographic variables: average household monthly net income (greater than or less than the Berlin average net monthly income), the lifetime prevalence of psychiatric disorders (current or history of clinically diagnosed psychiatric disorders), sex (male or female), and the mean age. The error bars in the mean age panel represent standard error.

Of the demographic predictors, the female sex robustly predicted membership to all three classes, such that there were greater odds of individuals with the female sex being in the most vulnerable class, followed by more vulnerable and then more resilient classes. Age also significantly predicted class membership, such that we observed greater odds of younger people being in the most vulnerable class, followed by the more resilient class, compared to the most resilient class. Furthermore, there were greater odds of individuals with lower-than-average levels of income being in more vulnerable and more resilient classes as compared to the most resilient class. Lastly, we also observed greater odds of individuals with a lifetime prevalence of psychiatric disorders being in a more vulnerable class compared to the most resilient. Meanwhile, civil status, employment status, years of education, and migration background were not significant predictors for class assignment. For an overview of the demographic characterization of the four classes, see Figure 5.

## 4. Discussion

The present study, embedded in the first phase of the CovSocial project [44], aimed to examine how mental health evolved over the course of the COVID-19 pandemic in Germany in 2020 and 2021, using a bi-polar resilience–vulnerability latent factor comprising multiple state self-report indicators, including both classic resilience and vulnerability aspects, such as stress, anxiety, depression, life satisfaction, optimism, but also more pandemic-specific fears and burdens. We had three main objectives. First, we aimed to chart out the general time course of resilience–vulnerability over seven timepoints during the pandemic in Germany, including two lockdowns, and additionally to understand the broader evolution of this trajectory in terms of acute stressor, recovery and pandemic fatigue effects. Second, we aimed to investigate whether there was heterogeneity in mental health responses concerning the various phases of the pandemic by examining the presence of different latent class trajectories of resilience-vulnerability. Lastly, we set out to understand whether inter-individual differences in trait characteristics, such as neuroticism and optimism, and demographic factors, such as sex and age, predicted mental health profiles during the pandemic to identify risk and protective factors.

Using data from the longitudinal CovSocial project, we found that resilience–vulnerability over the course of the pandemic, in a sample of 3522 Berliners, was indeed affected significantly by the dynamic evolution of the disease and the corresponding shifts in public health policy and related restrictions in 2020 and 2021. Importantly, we found evidence for the existence of three unique features of the dynamic resilience–vulnerability trajectories expanding over two main lockdowns in Germany. First, we observed an acute stressor effect as a result of the first lockdown following the declaration of the pandemic, which resulted in increased psychological vulnerability in March and April 2020 compared to the pre-pandemic time. This finding is in line with previous studies from across the globe that have shown an acute increase in mental health difficulties following the imposition of first social distancing and confinement measures [7,9,11]. Second, a recovery effect was noticed as the first lockdown was lifted in Germany, such that vulnerability decreased at the measurement occasion of June 2020, and individuals tended towards more resilient responses. This finding also corresponds with other studies that examined mental health trajectories during deconfinement periods, including from Germany, that found that individuals were overall showing more resilient responses during this period of time [11,13,14,15].

However, thirdly, a worrying “pandemic fatigue effect” was detected during the phase of highly dynamic public health policy changes in Germany from November 2020 to May 2021, which was characterized by worsening mental health and linearly increasing vulnerability with every passing month until peak vulnerability in March-April 2021. This finding provides evidence for a cumulative risk approach to the COVID-19 pandemic [30], such that increases in the degree of cumulative exposure to the pandemic-related restrictions led to further aggravation of psychological vulnerability. The finding also directly corroborates the conceptualization of a pandemic fatigue effect in the With:Resilience model [31]. Thus, while cross-sectional studies have linked decreased adherence to public health regulations to poor mental health during the pandemic [34,35], our present finding may provide direct empirical evidence of this mental health fatigue longitudinally. It is also in line with the few published studies that have shown magnified psychopathological symptoms in the long run as a result of extended lockdown measures [26,27]. Importantly, given that we measure resilience–vulnerability responses in the same time period (mid-March to mid-April) in the same sample, a direct descriptive comparison between the levels of vulnerability during 2020 and 2021 revealed that mental health became worse off from the first lockdown in 2020 to the next much longer lockdown a year later. These findings crucially extend prior work conducted in Germany that showed increasing improvements in mental health in the first few weeks of the pandemic [13] and found no changes in mental health difficulties in German students from the deconfinement period (July 2020) to the beginning of the second lockdown (November 2020) [63]. This suggests that when considering longer assessment periods under protracted stressors, such as the extended second lockdown in Germany, mental health declines palpably.

Moreover, we also observed heterogeneity in terms of resilience–vulnerability time course in our sample, i.e., the course of the pandemic affected different people differently. Accordingly, we detected four distinct latent classes of resilience–vulnerability trajectories in our sample: most vulnerable, more vulnerable, more resilient, and most resilient. This corresponds with prevalent models of resilience that postulate the emergence of heterogeneous trajectories of psychological well-being, coping, and resilience in response to stress [36,37]. However, the trajectories depicted by the four classes in the present data do not align with the hypothesized trajectories in the With:Resilience conceptual framework [31]. Specifically, we do not observe in our data the two rather flat resilience–vulnerability trajectories hypothesized in the With:Resilience framework (chronic vulnerability and non-reactive resilience), wherein individuals do not respond to the various phases of the pandemic. Instead, at first glance, all four classes seemed to be characterized by fairly similar temporal patterns of resilience–vulnerability during the course of the pandemic, all showing acute stressor and pandemic fatigue effects. This finding is a departure from prevailing resilience models focusing on the effects of stressors, such as individual challenging life events, traumatic events, or natural disasters [28,36,37,64]; it seems that the COVID-19 pandemic, and the related lockdowns, present a unique global stressor that left its mark on the psychological well-being of all segments of the population.

While nearly two-thirds of the participants in our sample fell into the more resilient classes (~70%) in line with other studies examining mental health response heterogeneity during the pandemic [65], we also identified two vulnerable groups that comprise nearly 30% of the sample. When looking at the descriptive trajectories found in our data, what becomes immediately evident is the relevance of pre-existing differences in resilience–vulnerability responses across classes. Since the classes differ in their baseline levels, it implies that the onset and evolution of the pandemic only served to exacerbate the pre-existing differences in mental health vulnerability depicted by different groups. Upon closer look at the four trajectories, the distinct responses of these classes to the various pandemic phases are also revealed. Those in the most and more vulnerable classes seemingly showed a more pronounced acute response to the first lockdown and less of a recovery in psychological well-being after the first lockdown, compared to the most and more resilient classes who seemed to show a return to pre-pandemic levels of resilience–vulnerability in June 2020. Moreover, the steepest rise in vulnerability during the second lockdown was depicted by the more vulnerable class, further supporting the idea of exacerbation of pre-existing mental vulnerability. As such, the latent class trajectory analysis hinted at the complex and specific nature of the impact that the pandemic had on different individuals.

In the next step focusing on the identification of risk and protective factors, the logistic regression revealed that, indeed, different trait and demographic aspects predicted which trajectory of resilience–vulnerability was exhibited by an individual. In line with diathesis-stress models and the With:Resilience model [31,66] and previous studies conducted during the pandemic [7,13,15,38,39], we found that trait psychological aspects, such as latent trait factors of resilience-vulnerability, adaptive coping capacities, and social belonging predicted class membership. Higher levels of trait vulnerability, comprising aspects such as chronic stress, neuroticism, and pessimism [51], were one of the strongest trait predictors of who fell into the most and more vulnerable classes. Higher levels of trait loneliness and lower levels of social support, as reflected in the trait social belonging factor, were also associated with individuals exhibiting the more vulnerable trajectory. This is an especially important finding given the socially isolating nature of the pandemic, and related lockdowns led to reports of significant increases in loneliness, especially amongst the young [63,67,68,69]. Interestingly, we found that higher levels of trait adaptive capacities factor, which includes the use of adaptive coping strategies, optimism, and self-compassion, predicted membership to the two middle classes in comparison to the most resilient class. Higher levels of adaptive capacities being predictors of more vulnerable and less resilient classes during the pandemic could be a function of the stressor itself. Given the confinement and social isolation restrictions, it is possible that people who could not implement their trait adaptive capacities to counter the stressful effects of the pandemic, such as using trait adaptive coping and regulation strategies like behavioral activation, ended up then showing a vulnerable response because they could not successfully cope with the stressors using the resources they normally would. Similarly, it has been argued that in the context of the COVID-19 pandemic, social factors that facilitate coping and adaptation may lead to both traumatic stress and posttraumatic growth [70]. This view aligns with prevalent psychopathology perspectives that posit implementation failure and not only access to a limited repertoire of adaptive coping capacities as one of the key mechanisms for heightened mental health problems [71,72]. As such, while these individuals might have the repertoire of adaptive capacities at hand to be able to cope well in other socially non-isolating contexts, their failure to implement these capacities in the pandemic context potentially led these individuals to fall into a more vulnerable or less resilient category. However, in comparison to the most vulnerable class, the more vulnerable and more resilient classes showed stronger recovery in June 2020, perhaps indicating a role of higher trait adaptive capacities.

Focusing on key demographic variables, we also found the female sex to be one of the strongest demographic predictors of being in the most and more vulnerable class. Similarly, age also turned out to be a significant predictor, with younger individuals having greater odds of depicting the most vulnerable trajectory. Both individuals with lower socioeconomic status (earning less than the Berlin net monthly average income) and individuals with a lifetime prevalence of psychiatric disorders had greater odds of exhibiting a more vulnerable trajectory. These findings indicate that mental health disparities that existed prior to the pandemic have perhaps been exaggerated in the pandemic context as the most and more vulnerable groups depicted stronger acute stressor and pandemic fatigue effects and showed muted recovery. Females, younger cohorts, and individuals in lower-income groups have consistently been shown to be at risk for developing mental health problems [73,74,75], and studies conducted during the pandemic have also shown similar patterns [39,76,77,78]. While we do not investigate the mechanisms that might underlie these disparities, they could potentially be a reflection of aspects such as increased childcare burden for women, decreased social contact with peers for younger people, or decreased job or financial security for individuals in lower income groups, that have been documented by other studies [78,79]. As such, it can be understood that mental health disparities in terms of sex, age, history of mental illnesses, and socioeconomic status increased as the lockdown measures became prolonged. This also implies that while the pandemic might have been theorized as a collective stressor, conceptually speaking, socially disadvantaged groups were affected by it differently. The findings from the current study accordingly present a nuanced and specified view of pandemic-related mental health impacts and extend related findings on collective stressors and existing social inequalities [80,81].

## 5. Strengths and Limitations

The present study is one of the rare studies to examine mental health trajectories during the multiple lockdowns in Germany in 2020 and 2021, tracking different resilience–vulnerability trajectories in a fairly large community sample (n = 3522) for a relatively long period of time (measurement occasions covering >12 months). Moreover, our assessment of resilience–vulnerability was not limited to specific aspects of mental health using only a single measure, but we endeavored to examine a wide range of vulnerability and resilience domains to provide a more comprehensive picture of mental health during the pandemic. Using latent modeling approaches to our advantage, we were able to uniquely assess very pandemic-specific vulnerability aspects, such as loneliness or pandemic-related burdens (e.g., burdens of care and finances), in addition to common mental health difficulties, such as depressive and anxious symptoms. We adopted a similar holistic approach to predictors of mental health trajectories, including not only classic trait aspects, such as neuroticism or pessimism, but also social trait capacities, such as empathy and trust.

Regarding representativeness, our sample was representative of the Berlin population in terms of average age (43 years in both our sample and the Berlin population [60]). While we had an overrepresentation of females in the sample (65.1%) in comparison to the Berlin population (50.5%), this is similar to other online psychology studies conducted during the pandemic both in Germany and globally [7,13,26,63,82]. Moreover, our sample was highly comparable to the German population in terms of the lifetime prevalence of psychiatric disorders. In our sample, 24.9% of individuals indicated a history of psychiatric disorders, and in the German population, this is 27.7% [83]. Similarly, the sample was comparable to the Berlin population in terms of married or registered partnerships cohabiting, which in our sample was 37%, and in the Berlin population, this was 34.2%.

Despite the strengths of this work, there are also crucial limitations that must be addressed. A key limitation of the current study is related to the retrospective assessment of the first three timepoints, which may have led to a recall bias. Given the unpredictable nature of the pandemic, retrospective study designs have been applied in prior work investigating longitudinal mental health trajectories during the pandemic [84,85,86]. The retrospective timepoints in the present study (January 2020, March–April 2020, and June 2020) were assessed after the easing of the first lockdown measures in Germany (11 September 2020 until October 2020). However, to ensure a more vivid recall of the feelings and behaviors in specific time periods, participants underwent a short perspective-taking exercise before answering questions for each of the three timepoints. In the perspective-taking exercise, participants were presented with a brief text that reminded them of the main national and international current events of political and societal importance taking place and being reported in local and national news at that time (see Supplement File S1). Participants were also periodically presented with this text prompt at several points in between the survey. Participants were asked to answer all questions taking the perspective of timepoint and events described in the text prompts. Additionally, participants also completed an additional question that assessed perspective-taking, indicating that it was not very difficult to recall specific periods retrospectively (mean = 2.92 ± 1.72, range = 0–8, higher scores represent more difficulties, missing n = 1049). Importantly, average values of pandemic-specific indicators, such as pandemic-related burdens and fears, showed very low values at the first assessment timepoint, as reported in a previous study from the CovSocial project [11]. Both these aspects more concretely attest that participants could indeed distinguish between the specific time periods when answering the questionnaires of the first three timepoints.

A further limitation of the present study is that the sample is not representative of the Berlin population in terms of average net monthly income and migration background. While in our sample, the average net monthly income was €3227, in the Berlin population, this number is €2175, indicating that the current sample, on average, had higher income than the larger population. Although in our study, we already detect significant effects of income levels on vulnerability, with lower-than-average income groups emerging to be a risk group for mental health. However, future studies employing even more stratified samples and further diverse socioeconomic groups could provide more precise insights into mental health patterns under sustained stressors. Moreover, while nearly 37% of Berliners reported having a migratory background in 2021 [60], in our sample, this number was only about 10% which is significantly less. The primary reason for this could be the inclusion criteria for the study requiring proficiency in the German language since the study was conducted exclusively in the German language. For conceptual clarity, migration background, as assessed in the Berlin micro-census, could be seen as the assessment of ethnic identity, i.e., it includes those with foreign nationality but also first-, second-, and even third-generation migrants to Germany. Migration background can be an important predictor of mental health [87], and future studies should employ more representative samples to assess the effect of interaction between pandemic stressors and migration status on mental health trajectories. Furthermore, migration background, as conceptualized by the Berlin census and correspondingly in this study, is treated as an umbrella factor, which makes it a rather flawed measure of racial and ethnic diversity [88]. Such an assessment of migration background necessarily excludes the differing predictive effects of race and ethnicity on mental health during the pandemic. A differentiated view, therefore, would be necessary to develop an even sharper understanding of mental health disparities. Therefore, future studies with more representative and fully random samples drawn from the Berlin population would be necessary. Lastly, while the present study delineates key risk groups, given the scope of the present study, we were unable to directly investigate the mechanisms that might underlie these disparities. Although, as mentioned previously, some other studies have linked these disparities to increased childcare burden, decreased social interaction with peers, and decreased job or financial security [78,79], a further detailed examination is necessary to provide a more nuanced view of the mechanistic processes.

## 6. Conclusions

The present work aimed to understand how different mental health trajectories evolved over the course of the pandemic in Germany in 2020 and 2021, including two lockdowns, and what factors served as protective or risk factors for psychological well-being. The strengths of the present work lie in the examination of mental health trajectories over a long period (>12 months) using a holistic range of measures covering resilience and vulnerability responses comprehensively. While there was evidence for an overall trajectory of resilience–vulnerability that was characterized by intermittent periods of acute stress after the first lockdown, recovery during re-opening, and pandemic fatigue during the longer second lockdown, we also observed significant heterogeneity and complexity of mental health response. A latent class analysis enabled the identification of four different classes of mental health trajectories that differed in the intensity of their responses to the various phases of the pandemic. While the two more vulnerable classes showed muted recovery after the first lockdown and more pronounced pandemic fatigue effects during the second lockdown, the two more resilient classes seemingly recovered to baseline levels of resilience after the first lockdown and showed a significantly milder increase in vulnerability in the second lockdown. An examination of various predictors of mental health trajectories revealed considerable disparities, such that females, younger people, those with a history of psychiatric illnesses, and individuals in lower income groups emerged as key risk groups, along with those who had high levels of trait psychological vulnerability and low levels of social belonging. The present study has helped us understand that when considering the longer-term view (>12 months) on mental health during the pandemic in Germany, covering various phases of lockdowns and easing of restrictions, the average response seems to have been characterized by increased mental health burdens and measurable pandemic fatigue effects. Focusing further on the evolution of different mental health trajectories also shows that the various phases of the pandemic in Germany have not been experienced uniformly, extending previous findings [13] and in line with recent conceptual frameworks [31]. Moreover, even in an economically developed country, females, young cohorts, those with a history of psychiatric illnesses, and socially disadvantaged groups, such as lower socioeconomic status individuals, remained the most vulnerable among us. Both governmental support as well as interventional efforts, specifically ones aimed at these vulnerable groups, will be necessary to avoid long-term pandemic-related deterioration [89] of mental well-being.

## Figures and Tables

**Table 1:** Demographic and COVID-19-related characteristics of the sample.

	Mean (SD)	Count (%)
Age	44 (12.7)	
Female		2293 (65.1)
Marital status		
Single		1709 (48.5)
Married		1302 (37)
Divorced		293 (8.3)
Other		218 (6.2)
Years of education	17 (3.9)	
Average monthly net household income in EUR	3227 (1210)	
Migration background	384 (10.9)	
Working situation		
Full-time		1937 (55)
Part-time		752 (21.4)
Retired		116 (3.3)
Unemployed		93 (2.6)
Other		624 (17.7)
Working hours per week; mean	35.7 (11)	
Diagnosed mental disorder in lifetime		876 (24.9)
Depressive disorder		652 (18.4)
Anxiety disorder		297 (8.4)
Trauma-related disorder		162 (4.6)
COVID-19		
Biological risk group		836 (23.8)
Job with heightened risk of infection		865 (25.2)

**Table 2:** Comparison of model fit, classification accuracy, and class distributions of the 1- to 5-class models. Class 1 always refers to the class with the highest level of vulnerability. AIC = Akaike’s Information Criterion, and BIC = Bayesian Information Criterion.

Number of Classes	AIC	BIC	Sample Size-Adjusted BIC	Log Likelihood	Entropy	Class 1 (N)	Class 2 (N)	Class 3 (N)	Class 4 (N)	Class 5 (N)
1	40,033.87	40,181.87	40,105.61	−19,802.49		100%				
2	36,392.66	36,657.84	36,521.20	−18,153.33	0.69	44.5%	55.5%			
3	35,832.02	36,214.36	36,017.35	−17,854.01	0.61	44.6%	35.5%	19.9%		
4	35,476.18	35,975.69	35,718.31	−17,657.09	0.644	13.0%	15.93%	46.11%	24.96%	
5	35,307.75	35,924.43	35,606.68	−17,553.87	0.66	10.2%	14.7%	42.9%	5.5%	25.6%

**Table 3:** The average posterior latent class probabilities of the 4-class mixture model. In the diagonal: the average probability of a participant being assigned to a class. In the off-diagonal: the average probability of being assigned to another class. Class 1 always refers to the class with the highest level of vulnerability.

		Most Likely Latent Class			
		1	2	3	4
Latent Class	1	0.830	0.088	0.000	0.001
	2	0.166	0.771	0.115	0.101
	3	0.000	0.055	0.777	0.059
	4	0.003	0.086	0.107	0.839

**Table 4:** Parameter estimates from the Growth Mixture Model per class. *p*-values in brackets. Significant estimates (*p* < 0.05) are in bold. Res-Vul T1 = State Resilience–vulnerability at time point 1, LCS = Latent change score. Intercept and slope refer to the linear growth curve from T4 to T7.

	Class			
	Most Vulnerable	More Vulnerable	More Resilient	Most Resilient
Means				
Res-Vul T1	**1.017** (<0.001)	0.173 (0.233)	0.013 (0.861)	**−0.720** (<0.001)
LCS1	**1.346** (<0.001)	**0.898** (<0.001)	**0.618** (<0.001)	**0.248** (<0.001)
LCS2	**−0.640** (<0.001)	**−0.585** (<0.001)	**−0.545** (<0.001)	**−0.324** (<0.001)
Intercept	**2.188** (<0.001)	**1.065** (<0.001)	**0.517** (<0.001)	**−0.474** (<0.001)
Slope	**0.044** (<0.001)	**0.073** (<0.001)	**0.061** (<0.001)	**0.040** (<0.001)
Variances				
Res-Vul T1	**1.455** (<0.001)	**0.922** (<0.001)	**0.530** (<0.001)	**0.281** (<0.001)
LCS1	**1.653** (<0.001)	**1.387** (<0.001)	**0.633** (<0.001)	**0.174** (<0.001)
LCS2	**0.862** (0.001)	**1.026** (<0.001)	**0.338** (<0.001)	**0.079** (<0.001)
Intercept	**0.525** (<0.001)	**0.820** (<0.001)	**0.582** (<0.001)	**0.477** (<0.001)
Slope	0.001 (0.558)	**0.029** (<0.001)	**0.005** (<0.001)	**0.003** (<0.001)
Covariances				
LCS1 with Res-Vul T1	**−1.157** (<0.001)	**−0.597** (0.042)	**−0.263** (<0.001)	**0.030** (<0.001)
LCS1 with LCS2	**−0.396** (0.004)	**−0.600** (<0.001)	**−0.269** (<0.001)	**−0.070** (<0.001)
LCS2 with Res-Vul T1	0.058 (0.428)	0.050 (0.372)	**0.062** (0.001)	−0.009 (0.263)
Slope with Intercept	−0.001 (0.826)	**−0.042** (0.010)	**−0.007** (0.022)	0.001 (0.697)

**Table 5:** The results of post-hoc comparisons with Wald’s *t*-test. *p*-values in brackets. Significant comparisons (*p* < 0.0083) in bold. LCS = latent change score.

	LCS1 Comparison	LCS2 Comparison	Slope Comparison
Class 1—Class 2	2.245 (0.134)	0.165 (0.685)	0.129 (0.720)
Class 1—Class 3	**15.420** (<0.001)	1.029 (0.310)	2.817 (0.093)
Class 1—Class 4	**27.565** (<0.001)	**11.471** (<0.001)	0.129 (0.720)
Class 2—Class 3	3.833 (0.050)	0.309 (0.579)	1.045 (0.307)
Class 2—Class 4	**34.214** (<0.001)	**17.087** (<0.001)	**8.815** (0.003)
Class 3—Class 4	**48.338** (<0.001)	**52.515** (<0.001)	6.404 (0.011)

**Table 6:** Results (odds ratios) from the multinomial regression analysis with predictors for the resilience–vulnerability trajectory classes, with the most resilient class as the reference group. CI = confidence interval; reference group = most resilient trajectory (n = 863); most vulnerable trajectory (n = 450); more vulnerable trajectory (n = 587); more resilient trajectory (n = 1622). * *p* < 0.05. ** *p* < 0.01. *** *p* < 0.001.

Predictor	Most Vulnerable Multinomial Odds Ratio (95% CI)	More Vulnerable Multinomial Odds Ratio (95% CI)	More Resilient Multinomial Odds Ratio (95% CI)
Trait Resilience-Vulnerability	884.19 (323.11, 2419.55) ***	23.29 (15.30, 35.44) ***	18.71 (13.18, 26.58) ***
Trait Adaptive Capacities	1.27 (0.65, 2.51)	2.37 (1.63, 3.45) **	1.62 (1.19, 2.20) *
Trait Social Belonging	0.83 (0.49, 1.39)	0.66 (0.51, 0.87) *	0.97 (0.76, 1.23)
Trait Social Capacities	1.11 (0.61, 2.01)	0.95 (0.67, 1.32)	0.83 (0.63, 1.08)
Age	0.96 (0.93, 0.99) *	0.98 (0.96, 1.001)	0.98 (0.96, 0.99) *
Female Sex	3.26 (1.22, 8.75) ***	3.97 (2.45, 6.43) ***	2.79 (1.96, 3.99) ***
History of Psychiatric Diagnosis	2.12 (0.79, 5.62)	2.14 (1.21, 3.79) **	1.43 (0.84, 2.44)
Married/Cohabiting/Partnership	1.15 (0.85, 1.54)	1.12 (0.93, 1.34)	1.10 (0.95, 1.28)
Lower than average household income	0.98 (0.91, 1.05)	0.95 (0.92, 0.99) *	0.96 (0.93, 0.99) *
No full-time employment	0.97 (0.88, 1.08)	0.96 (0.91, 1.01)	0.96 (0.92, 1.01)
Years of Education	0.99 (0.86, 1.14)	1.05 (0.99, 1.11)	1.00 (0.96, 1.04)
Migration background	3.23 (0.85, 12.28)	1.61 (0.85, 3.06)	1.47 (0.82, 2.64)

## Data Availability

Data will be made available upon request.

## Supplementary Materials

The following supporting information can be downloaded at: https://www.mdpi.com/article/10.3390/healthcare11091305/s1, File S1: Recruitment, Dropouts, and Outlier Detection; File S2: Measures; File S3: Mean values of measures T1–T7.
