# Supplementary material for: Heterogeneous Mental Health Responses to the COVID-19 Pandemic in Germany: An Examination of Long-Term Trajectories, Risk Factors, and Vulnerable Groups
Source: Healthcare (Basel). 2023 May 3;11(9):1305. doi: 10.3390/healthcare11091305 (PMC10177770; doi:10.3390/healthcare11091305)
Supplement: Supplementary file 1 [file healthcare-11-01305-s001.zip › File S2.pdf]

<u>**Supplement S2**</u>

Measures

Several state constructs were measured using validated scales, while others were assessed through self-generated questions. The following variables of state vulnerability were assessed: emotional state (valence and arousal), alcohol use and control of alcohol use, negative news consumption, stress and perceived stress, internet consumption, compulsive internet use, craving for internet use, strains and burdens, loneliness, depressive symptoms, anxiety symptoms, psychosomatic difficulties, aggression, and perceived appropriateness of lockdown and protective measures. The following variables of state resilience were assessed: self-efficacy, optimism, seeing crisis as an opportunity (Positive Reappraisal), life satisfaction, resilience, and the use of coping strategies. Please see Table 1 for all the scales and self-generated questions administered to participants. Participants completed the same state measures seven times during the study for each of the seven timepoints, January 2020 (Pre-lockdown period; T1), Mid-March to Mid-April 2020 (Lockdown period; T2), and June 2020 (Post-Lockdown period; T3), October 2020 (“Lockdown light”; T4), and three timepoints November 2020 (T5), December 2020 (T6) and March-April 2021 (T7) over the “hard lockdown”.

The state measures for T1-T3 were completed retrospectively during the period of 11 September to 7 December 2020. Therefore, in order to ensure that participants were completely mentally immersed into the timeframe of the particular timepoint of interest, they underwent a brief perspective-taking exercise prior to responding to the state-related measures. Prior to each block of the state questionnaires, participants were asked to take a moment to recollect what was happening during the timepoint. They were reminded of the major world events taking place at that particular timepoint, and were asked to recall the major individual life events taking place during that time. They were also asked to consult their planners, schedules and agendas to jog their memory of the daily life events taking place during the period, and to immerse themselves in the perspective of the particular timepoint. For example, prior to completing the state measures for T1, participants were reminded about the recent Christmas and New Year’s Eve celebrations of 2019, major local Berlin events such as arrival of the baby panda bear twins at the Berlin Zoo, and world events such as World Economic Forum at Davos and the Australian bushfires of January 2020. They were then asked to consult their personal planners for the month of January 2020 to remind themselves of the daily events taking place in their lives during that month. Once the participants had appropriately immersed themselves into the perspective of the timepoint, they could then begin completing the state measures from that perspective. The perspective-taking exercise presented for each of the three timepoints is provided below.

Perspective-taking Exercise:

These perspective-taking exercises were performed prior to participants completing the retrospective questionnaires for T1, T2, T3.

T1 – Pre-lockdown period.

The first phase covers the period before the acute corona crisis and relates to the month of January 2020

[January 2020 (before to the crisis)]

The following questionnaire relates to the phase in January 2020, and thus to the time frame prior to the acute corona crisis.

Please take a moment to remember this period. You recently celebrated Christmas and New Year’s Eve and you have just started into the year 2020. Newspapers reported about the bushfires in Australia, the climate demonstrations related to the World Economic Forum in Davos and the panda bear twins in the Berlin Zoo. Please think specifically about personal events in January 2020, as well as any regular activities or trips you did during that period. Also, please review the expectations and plans you had at the start of the year 2020. Ideally, you could take a look into your personal planner to recall personal appointments or meetings to put yourself into said time period as best as possible.

T2 – Lockdown period.

The second phase is defined as the period of lockdown and refers to the month between mid-March and mid-April 2020

• The third phase describes the timeframe of relaxation of the restrictions and refers to the month of June 2020

[mid-March – mid-April 2020 (lockdown)]

The following questionnaire refers to the lockdown phase between mid-March and mid-April 2020.

Please take a moment to remember this period. After carnival meetings in North Rhine-Westphalia, the coronavirus has also spread faster and faster in Germany. News from Italy and Spain showed long lines in front of supermarkets and overcrowded hospitals. In Germany, schools, stores, restaurants, etc. were closed. Public life came to a standstill for the most part. Angela Merkel addressed the population in a televised speech and called on people to stay at home if possible. Please think specifically about personal events in mid-March and mid-April 2020, as well as any regular activities or trips you did during that period. Ideally, you could take a look into your personal planner to recall personal appointments or meetings to put yourself into said time period as best as possible.

T3 – Re-opening period.

The third phase describes the timeframe of relaxation of the restrictions and refers to the month of June 2020

[June 2020 (relaxations)]

The following questionnaire refers to the phase of relaxations in June 2020.

Please take a moment to remember this period. Stores, hairdressers as well as gastronomic establishments gradually reopened. Public life resumed with restrictions, such as the mask requirement. Anti-racism protests following the death of George Floyd took place in Germany and elsewhere. COVID-19 broke out on a large scale at the Tönnies slaughterhouse in Westphalia, and the Corona warning app was released. Berlin schools started their summer vacations and some headed off on vacation. Now please also think about personal events in June 2020 as well as regular activities or trips you pursued during this period. It is best to look at your calendar again to recall private appointments or encounters and thereby put yourself in this time as well as possible.

Table S1. State measures. The table below provides an overview of the scales and self-generated questions administered to participants as part of state measures assessment.

Construct	Short	Questions	Items	Respondence scale	Reference
Life satisfaction	Lezu_state	How satisfied were you with your life?		0 (dissatisfied) – 8 (very satisfied)	Self-generated
Optimism	Optimismus_state	How optimistic were you?		Slider:<br>0 (not at all) – 8 (highly optimistic)	Self-generated
Burdens	Belastung	How burdened did you feel by the following factors?	Negative political events and media coverage	Slider: 0 (not at all) – 8 (highly burdened)	Self-generated
			Financial or economic problems		
			Loss of job or insolvency of a private company for yourself or someone in your household		
			Problems with the supply of basic needs or the procurement of goods and services		
			Personal physical health problems		
			Physical health problems of friends, family members or colleagues		
			Personal mental health problems		
			Mental health problems of friends, family members or colleagues		
			Personal increased risk of catching an infectious disease		
			Increased risk of an infectious disease among friends, family members or colleagues		
			Problems concerning childcare, difficulties in coordinating childcare and work		
			Tension or conflicts at home or family conflicts		
			Tension or conflicts with friends and / or acquaintances		
			Stressful experiences (e.g. conflicts) at work, university or other occupation		
			Increased workload		
			Housing conditions such as tight living space (e.g. no access to a private outdoor area, too many roommates)		
			Loss of a loved one through separation or death		
			Limitations of social contacts and / or opportunities to participate in important social events		
			Limited travel options (private or business)		
			Limited leisure opportunities and / or little exercise		
			Discrimination based on your migration status		
			Discrimination based on your sexual orientation		
			Discrimination based on your gender		
			Other reasons		
Stress	Perceived Stress Scale (PSS4)			(PSS-4; Cohen, Kamarck & Mermelstein, 1983)	
Self-efficacy	Allgemeine Selbstwirksamkeit Kurzskala (ASKU)			(ASKU; Beierlein et al., 2013)	
			… backpain?		
			… exhaustion / tiredness?		

Psychosomatic	Psychosomatik	How much did you suffer from …	… sleep disorders?	Slider: 0 (not at all) – 8 (very much)	Self-generated
			… headaches / migraine?		
			… cardiovascular problems?		
			… gastrointestinal complaints		
			… cold symptoms (e.g. throat-, nose- und ear pain, cough, etc.)?		
Depression	Patient Health Questionnaire (PHQ2)			(PHQ-2; Löwe, Kroenke & Gräfe, 2005)	
Loneliness	Einsamkeit	How lonely did you feel?		Slider: 0 (not at all) – 8 (very lonely)	Self-generated
Crisis as chance (positive reappraisal)	Chance	Please evaluate the following statements.	Crises or problems have also brought positive changes for me personally.	Slider: 0 (not true at all) – 8 (perfectly true)	Self-generated
			Crises and problems have also brought positive changes for society in general.		
Coping	Coping	How much have the following things helped you to overcome crises and problems?	Support from others	Slider: 0 (not at all) – 8 (very much)	Self-generated
			Physical exercise		
			Spend time in nature		
			Humour		
			Religion, spirituality, meditation		
			Cultural offerings (also online)		
			Music		
			Actively changing the situation		
			Trying to accept the situation		
			Distraction		
			Open display of emotions when you are not doing well		
			Mentally plan the next steps ahead		
Anxiety	Ängstlich	How anxious were you?		Slider: 0 (not at all) – 8 (very anxious)	Self-generated
	Generalized Anxiety Disorder Scale (GAD-2)			(GAD-2; Kroenke et al., 2007)	
Stress recovery	Widerstand_schnell	How quickly did you recover after demanding / stressful moments?		Slider: 0 (very slowly) – 8 (very quickly)	Self-generated
	Widerstand_leicht	How difficult was it for you to recover from demanding / stressful situations?			
