# Supplementary material for: Heterogeneous Mental Health Responses to the COVID-19 Pandemic in Germany: An Examination of Long-Term Trajectories, Risk Factors, and Vulnerable Groups
Source: Healthcare (Basel). 2023 May 3;11(9):1305. doi: 10.3390/healthcare11091305 (PMC10177770; doi:10.3390/healthcare11091305)
Supplement: Supplementary file 1 [file healthcare-11-01305-s001.zip › File S3.pdf]

Supplement S3

Table S2. Mean values of indicators T1–T7. The following table provides the mean values of all the indicators that entered the resilience-vulnerability latent factors at each of the seven timepoints.

	**T1** Mean (SD)	**T2** Mean (SD)	**T3** Mean (SD)	**T4** Mean (SD)	**T5** Mean (SD)	**T6** Mean (SD)	**T7** Mean (SD)
**Loneliness [0-8]**	1.85 (2.08)	3.24 (2.52)	2.24 (2.20)	3.28 (2.49)	3.39 (2.52)	3.68 (2.52)	3.79 (2.55)
**Anxious**	1.94 (1.89)	3.67 (2.30)	2.49 (2.07)	3.10 (2.28)	3.09 (2.25)	3.06 (2.28)	3.20 (2.34)
**Depression (PHQ-2)**	0.67 (0.62)	0.95 (0.76)	0.74 (0.69)	0.96 (0.77)	0.97 (0.77)	1.06 (0.81)	1.09 (0.82)
**Anxiety (GAD-2)**	0.62 (0.66)	0.92 (.80)	0.71 (0.72)	0.86 (0.78)	0.82 (0.76)	0.87 (0.82)	0.93 (0.83)
**Perceived Stress (PSS-4)**	1.47 (0.69)	1.77 (0.76)	1.51 (0.76)	1.67 (0.74)	1.66 (0.76)	1.72 (0.79)	1.79 (0.81)
**Stress [0-8]**	3.99 (2.07)	4.59 (2.29)	3.91 (2.14)	4.71 (2.13)	4.50 (2.15)	4.61 (2.21)	4.88 (2.13)
**Burdens [0-8]** Mean over 24 items*	1.38 (0.93)	2.26 (1.06)	1.82 (1.01)	2.17 (0.99)	2.15 (0.98)	2.17 (1.01)	2.33 (1.03)
**Psychosomatic Complaints [0-8]** Mean over 8 items**	1.77 (1.26)	1.93 (1.43)	1.52 (1.28)	2.26 (1.47)	2.20 (1.47)	2.22 (1.49)	2.42 (1.56)
**Resistance [0-8]**	5.03 (1.65)	4.60 (1.87)	4.92 (1.83)	4.34 (1.93)	4.32 (1.95)	4.12 (1.97)	4.01 (2.00)
**Life Satisfaction [0-8]**	5.76 (1.63)	4.66 (1.94)	5.41 (1.71)	4.94 (1.83)	4.85 (1.87)	4.58 (1.94)	4.43 (1.95)
**Optimism [0-8]**	5.69 (1.72)	4.33 (1.91)	5.03 (1.82)	4.05 (1.98)	4.13 (1.98)	3.88 (2.02)	3.69 (2.08)
**Chance [0-8]**	4.10 (1.95)	4.09 (2.04)	4.07 (2.05)	3.78 (2.02)	3.75 (2.04)	3.68 (2.04)	3.53 (2.08)

**Self-efficacy (ASKU)**	4.37 (0.72)	4.04 (0.89)	4.18 (0.83)	4.14 (0.84)	4.09 (0.86)	4.03 (0.88)	3.96 (0.93)
**Coping [0-8]** Mean over 12 items***	4.49 (1.18)	4.22 (1.16)	4.38 (1.17)	4.07 (1.19)	4.01 (1.22)	3.91 (1.26)	3.91 (1.24)

*The following items were assessed with the question "how burdened did you feel by the following?": Negative political events and media coverage, Financial or economic problems, Loss of job or insolvency of a private company for yourself or someone in your household, Problems with the supply of basic needs or the procurement of goods and services, Personal physical health problems, Physical health problems of friends, family members or colleagues, Personal mental health problems, Mental health problems of friends, family members or colleagues, Personal increased risk of catching an infectious disease, Increased risk of an infectious disease among friends, family members or colleagues, Problems concerning childcare, difficulties in coordinating childcare and work, Tension or conflicts at home or family conflicts, Tension or conflicts with friends and / or acquaintances, Stressful experiences (e.g. conflicts) at work, university or other occupation, Increased workload, Housing conditions such as tight living space (e.g. no access to a private outdoor area, too many roommates), Loss of a loved one through separation or death, Limitations of social contacts and / or opportunities to participate in important social events, Limited travel options (private or business), Limited leisure opportunities and / or little exercise, Discrimination based on your migration status, Discrimination based on your sexual orientation, Discrimination based on your gender, and Other Reasons.

**The following items were assessed with the question "How much did you suffer from?": Backpain, exhaustion / tiredness, headaches / migraine, cardiovascular problems, gastrointestinal complaints, and cold symptoms (e.g., throat-, nose- and ear-pain, cough, etc.).

***The following items were assessed with the question "How much have the following things helped you to overcome crises and problems?": Support from others, Physical exercise, Spend time in nature, Humour, Religion/spirituality/meditation, Cultural offerings (also online), Music, Actively changing the situation, Trying to accept the situation, Distraction, Open display of emotions when you are not doing well, and Mentally plan the next steps ahead.
